# The Desensitized Channelrhodopsin‐2 Photointermediate Contains 13 ‐*cis*, 15 ‐*syn* Retinal Schiff Base

**DOI:** 10.1002/anie.202015797

**Published:** 2021-06-17

**Authors:** Johanna Becker‐Baldus, Alexander Leeder, Lynda J. Brown, Richard C. D. Brown, Christian Bamann, Clemens Glaubitz

**Affiliations:** ^1^ Institute of Biophysical Chemistry and Centre for Biomolecular Magnetic Resonance Goethe University Frankfurt Max-von-Laue-Str. 9 60438 Frankfurt Germany; ^2^ Department of Chemistry University of Southampton Southampton SO17 1BJ UK; ^3^ Max-Planck-Institute of Biophysics Max-von-Laue-Str. 3 60438 Frankfurt Germany

**Keywords:** channelrhodopsin, dynamic nuclear polarization, membrane proteins, photocycle, solid-state NMR spectroscopy

## Abstract

Channelrhodopsin‐2 (ChR2) is a light‐gated cation channel and was used to lay the foundations of optogenetics. Its dark state X‐ray structure has been determined in 2017 for the wild‐type, which is the prototype for all other ChR variants. However, the mechanistic understanding of the channel function is still incomplete in terms of structural changes after photon absorption by the retinal chromophore and in the framework of functional models. Hence, detailed information needs to be collected on the dark state as well as on the different photointermediates. For ChR2 detailed knowledge on the chromophore configuration in the different states is still missing and a consensus has not been achieved. Using DNP‐enhanced solid‐state MAS NMR spectroscopy on proteoliposome samples, we unambiguously determined the chromophore configuration in the desensitized state, and we show that this state occurs towards the end of the photocycle.

## Graphical abstract

Channelrhodopsin‐2, a light driven cation channel with a retinal chromophore, undergoes a cyclic photoreaction with several photointermediates. Here, we analyze the chromophore configuration of the intermediates, which can be cryo‐trapped, in detail by DNP‐enhanced solid‐state NMR spectroscopy and reveal that the desensitized state is in a 13‐*cis*,15‐*syn* configuration. Our results also shed further light on the photocycle of the protein.

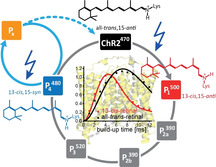

## Introduction

Microbial rhodopsins are heptahelical membrane proteins with a retinal chromophore covalently bound to a conserved lysine in helix G. A large variety of light‐driven functions is carried out by this protein family comprising proton and ion pumps, channels as well as sensors.^[1]^ In the dark‐adapted state the chromophore is either purely in the all‐*trans*,15‐*anti* configuration or a mixture of configurations is observed. Some microbial rhodopsins show light‐adaption which refers to light‐induced changes in the protein that remain even after the light has been switched off for some time. Usually, the function of the protein is conveyed by the photoreaction that starts from the all‐*trans*,15‐*anti* chromophore configuration. Illumination leads to retinal isomerization around the C13=C14 double bond resulting in a 13‐*cis*,15‐*anti* configuration in the first photointermediate. The system relaxes then via several photointermediates to the initial dark state with the all‐*trans*,15‐*anti* chromophore. The details of this photocycle vary depending on the protein and its function. Knowing which (photo)‐intermediates are adopted during the photocycle is a prerequisite for understanding the protein function as this varies between the different types of microbial rhodopsins.

Here, we focus on Channelrhodopsin‐2 from *Chlamydomonas reinhardtii* (ChR2).^[2]^ ChR2 is a cation channel and has found wide spread application in optogenetics.^[3]^ A crystal structure of the dark state of ChR2 has been determined but not of the photointermediates so far.^[4]^ These were investigated though with different spectroscopic techniques providing information about the changes of the protein and the chromophore compared to the initial dark state. In the dark‐adapted state (ChR2^470^), the protein has an absorption maximum at 470 nm and the retinal Schiff base chromophore is in the all‐*trans*,15‐*anti* configuration.^[5]^ Upon illumination, initial photoisomerization of the C13=C14 bond leads to the first photointermediate (P_1_^500^). Schiff base de‐protonation of P_1_^500^ results in at least two deprotonated states (P_2a_^390^ and P_2b_^390^) and is accompanied by channel opening.^[6]^ The channel remains open during Schiff base re‐protonation (P_3_^520^) but closes before the initial dark state is reached again. During continuous illumination, desensitization of the protein is observed and full photocurrents are only recovered after prolonged time in the dark.^[2]^ This is unique to channelrhodopsins in the microbial retinal family and usually unwanted in optogenetic applications. Understanding the channelrhodopsin photocycle in general and desensitization in particular is therefore of general interest to the biophysical community.

Desensitization is associated with the non‐conducting P_4_^480^ photointermediate which itself is photo active.[^5^, ^7^] No consensus has been achieved with respect to the position of P_4_^480^ in the photocycle and its chromophore configuration. Based on FT‐IR spectroscopy it was concluded that P_4_^480^ contains an all‐*trans*,15‐*anti* chromophore whereas a Resonance Raman study indicated that the chromophore configuration of P_4_^480^ is 13‐*cis*,15‐*syn*.^[8]^ In this work,^[8b]^ it was also postulated that P_4_^480^ is generated directly from the dark state via a photo reaction. This is in contrast to a previously published photocycle model in which P_4_^480^ is only formed during the open state decay.^[7]^ A comparison of the different models and P_4_^480^ configurations is given in Figure S1.

**Figure 1:**
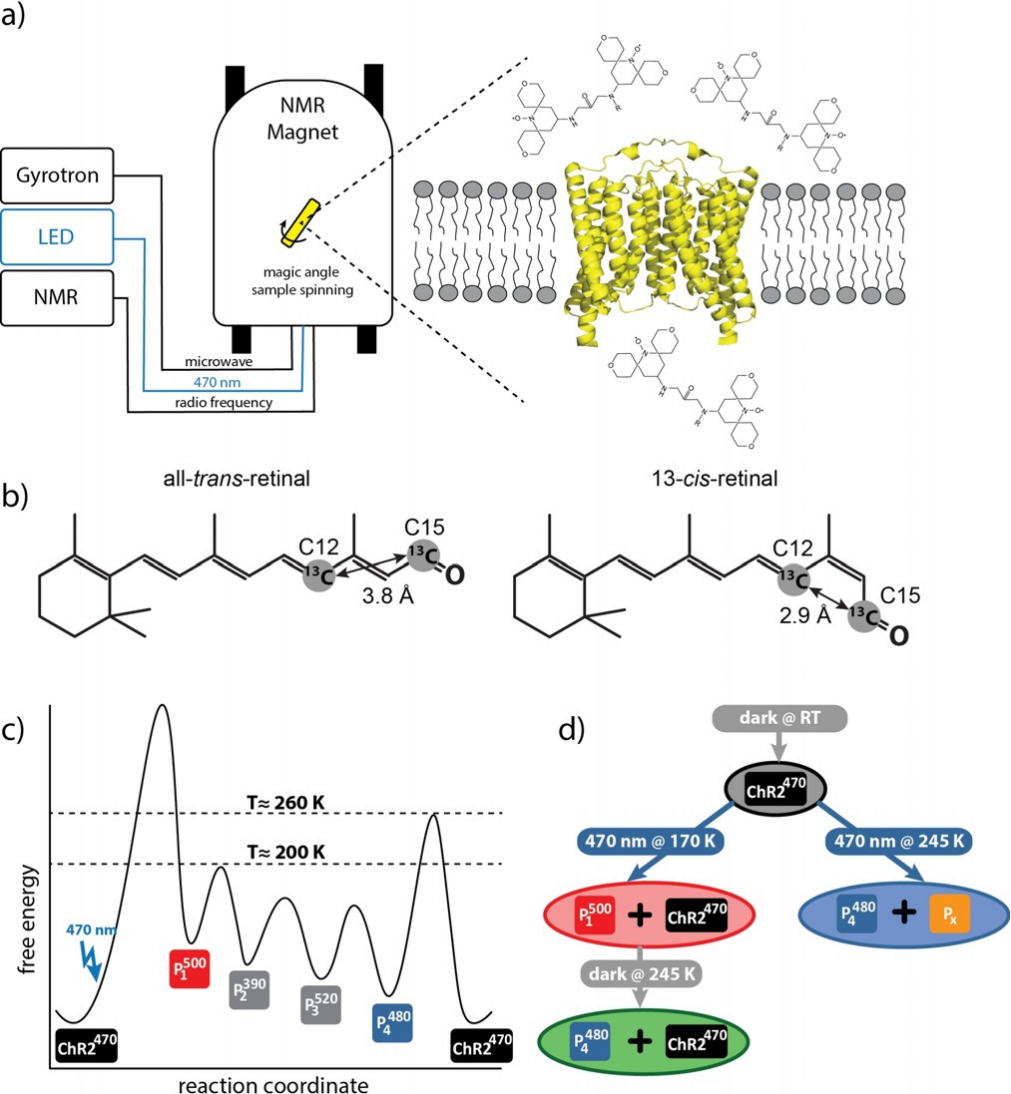
a) Cartoon of the experimental setup used in this work. Microwave, optical and radiofrequency irradiation from a gyrotron, an LED and the NMR console, respectively, can be applied simultaneously to the sample spinning with 8 kHz at the magic angle at cryogenic conditions. The sample consists of a proteoliposome pellet containing channelrhodopsin‐2, PDB code 6EID,^[4]^ which is surrounded by the polarizing agent AMUPol. b) Illustration of the isotope labelling Scheme and comparison of the C12 and C15 distances in all‐*trans*‐retinal and 13‐*cis*‐retinal. Distances are taken from the X‐ray structures of crystalline all‐*trans*‐retinal and 13‐*cis*‐retinal,^[12]^ respectively. [12,15‐^13^C_2_]‐all‐*trans*‐retinal was used to prepare the ChR2 sample. c) Schematic free energy landscape of the channelrhodopsin‐2 photocycle. The first photointermediate P_1_^500^ is formed after photoexcitation of ChR2^470^ and is stable at temperatures below ≈200 K. The following P_2_^390^ and P_3_^520^ states cannot be trapped as they decay already below 200 K and P_4_^480^ is reached. P_4_^480^ is stable below ≈260 K and returns to ChR2^470^ above this temperature. d) Illumination Scheme for the differently trapped states. Keeping the sample in the dark results in the pure ground state (ChR2^470^). Depending on the temperature, different states are populated by 470 nm illumination. At 170 K a mixture between P_1_^500^ and ChR2^470^ is generated and at 245 K, a mixture between P_4_^480^ and P_x_ is obtained. Heating the sample which was illuminated at 170 K to 245 K in the dark causes thermal relaxation and leads to a mixture of P_4_^480^ and ChR2^470^. The color of the circles corresponds to the color of the respective NMR‐spectrum in Figure 2.

To resolve these contradicting views, it is necessary to characterize the chromophore in the desensitized state of ChR2 in detail. In principle such information can be obtained from crystallographic data. However, to distinguish different chromophore configurations the resolution has to be extremely high and conditions have to be found under which the photointermediate can be studied. From the dark ChR2 X‐ray structure, the configuration of the chromophore could not be determined. Retinal extraction experiments and Resonance Raman spectroscopy had hinted towards a mixture of all‐*trans*,15‐*anti* and 13‐*cis*,15‐*syn* chromophore.^[9]^ However, the first technique is invasive and the second often suffers from problems with band assignments. Using solid‐state NMR spectroscopy of ChR2 in lipid bilayers circumvents both problems. Making use of signal‐enhancement by dynamic nuclear polarization, we and others could unambiguously show that in the dark the chromophore is purely in the all‐*trans*, 15‐*anti* configuration.[^5^, ^10^] With this technique it is also possible to study the chromophore in photointermediate states as long as they can be cryo‐trapped as demonstrated by us before.^[5]^ As low temperatures are required for cryo‐trapping and a high detection sensitivity is desired, the experiments are ideally suited for sensitivity‐enhanced MAS‐NMR spectroscopy based on dynamic nuclear polarization (DNP).^[11]^ In Figure 1 a our experimental setup is shown where DNP enhanced solid‐state NMR has been combined with in situ sample illumination in the optical range.

Cryo‐trapping of a photointermediate is possible when the energy barrier for its generation is lower (or can be overcome by photoexcitation) than the energy needed for its decay. We have visualized this in Figure 1 c for ChR2 with a free energy diagram based on our previous work.^[5]^ The photointermediate that can be trapped upon illumination at temperatures between 100 and 190 K was assigned to P_1_^500^ and always occurs in a mixture with ChR2^470^. This assignment was confirmed by optical spectroscopy under cryogenic condition. Another photointermediate can be generated by rising the temperature above 200 K. The same photointermediate, in a mixture with the dark state, is obtained by freeze quenching a sample that has been continuously illuminated at room temperature. This intermediate was assigned to P_4_^480^ as it is the state that will be enriched during continuous illumination due to its long life time. The same NMR signals are obtained when illuminating the sample at 245 K. Interestingly, in this case the ground state population is completely depleted but additional signals occur. This new photo intermediate is a photo product of P_4_^480^. As it is not known to which of the several postulated P_4_^480^ photo intermediates the cryo‐trapped intermediate corresponds, it was termed P_x_.

The ^13^C chemical shifts of the retinal Schiff base chromophore are very sensitive to the configuration of the chromophore: The C12 chemical shift is a readout for the configuration about the C13=C14 bond whereas the C14 chemical shift is directly related to the C15=N bond configuration.^[13]^ C12 and C14 are significantly shielded in the 13‐*cis* and 15‐*syn* configurations, respectively. In order to resolve any ambiguity about the chromophore configuration in the photointermediates, we analyzed their chemical shifts, and we determined the distance between C12 and C15, which is significantly shorter in the 13‐*cis* compared with the all‐*trans* configuration (Figure 1 b). In addition, we performed a thermal relaxation experiment to elucidate the position of P_4_^480^ within the photocycle. All experimental details are given in the SI.

## Results and Discussion

**Figure 2:**
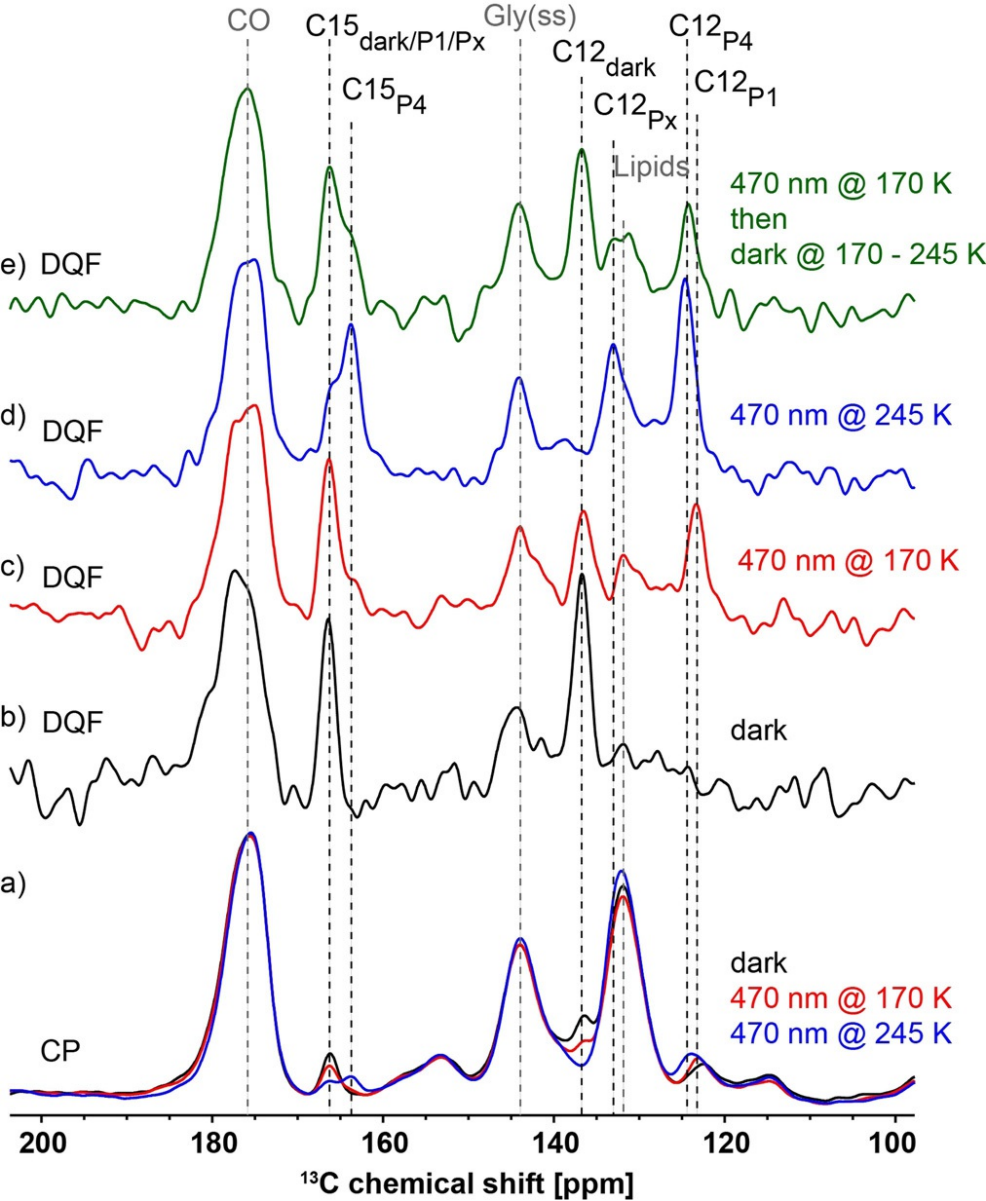
a) Cross polarization (CP) and b–e) SR26 double quantum filtered (DQF) spectra of [12,15‐^13^C_2_]‐all‐*trans*‐retinal‐ChR2 recorded after application of different illumination schemes: dark (black), 470 nm illumination at 170 K (red), 470 nm illumination at 245 K (blue), 470 nm illumination at 170 K followed by a temperature increase in the dark to 245 K (green), see Figure 1 b. Natural abundance background signals are labelled in grey (CO: carbonyl of protein and lipids, Gly(ss): Spinning side band of the glycerol signal and Lipids: olefinic lipid atoms) and [12,15‐^13^C_2_]‐retinal signals in black.

For detailed analysis of the retinal Schiff base chromophore in ChR2, [12,15‐^13^C_2_]‐all‐*trans*‐retinal‐ChR2 proteoliposomes were prepared. The proteoliposomes were doped with the radical AMUPol to enable DNP‐enhanced NMR experiments.^[14]^ The obtained signal enhancement was 50 (Figure S2). The associated dramatic improvement turned out to be crucial for the NMR experiments on the cryo‐trapped sample in order to deconvolute the signals of the different photointermediates. Furthermore, the long‐distance double quantum filtered experiments described below would not have been possible without the enhancement provided by the DNP experiment. ^13^C‐cross polarization spectra were recorded using three different illumination schemes: Without exposure to light, 470 nm illumination at 170 K and 470 nm illumination at 245 K (Figure 1 d). The spectra are shown in Figure 2 a and clear differences are observed around 165 ppm, 136 ppm, 132 ppm and 124 ppm. However, the spectra are dominated by the natural abundance contribution from the protein, the lipids and spinning side bands of the glycerol signal. Double quantum filtering should be able to suppress these signals. The large distance between ^13^C12 and ^13^C15 and the large chemical shift anisotropy (CSA) of these atoms make these experiments challenging. The standard Post‐C7 experiment used in our previous study for a one bond distance did not work for this sample.[^5^, ^15^] Therefore, we resorted to the CSA compensated SR26 sequence.^[16]^ This experiment, to our knowledge, has so far been applied only to small molecules at ambient temperature. Here we show that DNP‐enhanced solid‐state NMR enables the application of this sequence to a pair of quite distant atoms in the chromophore incorporated in a membrane protein. In addition, we could also quantify these long distances. Although some natural abundance signal intensity remains, the ^13^C12 and ^13^C15 signals can be much better identified in the spectra from the SR26 experiment compared to the CP spectra (Figure 2 b–d), especially in the region around 123 ppm which shows a background signal in the CP spectra which overlaps with the ^13^C12 signal in the spectra of the illuminated samples. The assignment of the ^13^C12 and ^13^C15 signals in the spectra is described in the SI and given in Table 1 together with the assignment of ^13^C14 which was obtained in our earlier work and recalled here to aid the analysis of the chromophore configuration.^[5]^

**Table 1:** Selected ^13^C‐retinal chemical shifts of the retinal chromophore in ChR2 and its photointermediates. A detailed description of the assignment is given in the SI.

State	^13^C12 [ppm]	^13^C14 [ppm]^[a]^	^13^C15 [ppm]
ChR2^470^	136.8	126.0	166.5
P_1_^500^	123.2	124.2	not resolved
P_4_^480^	124.5	119.3	163.8
P_x_	132.8	122.7	166.1

[a] ^13^C14 chemical shifts were taken from our previous publication.^[5]^

The ^13^C12 chemical shift clearly changes (−13.6 ppm) when trapping the first photointermediate, P_1_^500^, as expected for the isomerization around the C13=C14 bond. The effect on the ^13^C14 chemical shift in this intermediate is only minor showing that the C15=N bond remains in an *anti*‐configuration. Interestingly, the desensitized state, P_4_^480^, shows a very similar chemical shift for ^13^C12 compared with P_1_^500^ indicating a C13=C14 *cis*‐configuration in P_4_^480^. In addition, the ^13^C14 chemical shift shows a large shielding, which is expected for a C15=N *syn*‐bond. P_x_, the photointermediate of P_4_^480^, also shows an increased shielding of ^13^C12 and ^13^C14 but the effect is smaller compared to P_4_^480^ and interpretation is more ambiguous.

**Figure 3:**
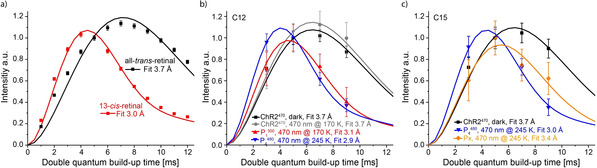
a) Double quantum build‐up curves for [12,15‐^13^C_2_]‐all‐*trans* retinal and [12,15‐^13^C_2_]‐13‐*cis*‐retinal in frozen solution. b) Double quantum build‐up curve of the ^13^C12 and c) of the ^13^C15 signals of [12,15‐^13^C_2_]‐retinal‐ChR2 recorded following the different illumination protocols shown in Figure 1 d. The best fits of the build‐up curves are shown together with the corresponding C12‐C15 distances. The error ranges of the fits are shown in Figure S5.

As additional support for the C13‐*cis* configuration in P_4_^480^ and to further elucidate the chromophore configuration in P_x_, we wanted to take advantage of the sensitivity of the ^13^C12‐^13^C15 distance to the C13=C14 configuration. This distance is shorter in the C13=C14‐*cis* confirmation (2.9 Å) than in the C13=C14‐*trans* configuration (3.8 Å) and can be used to distinguish them (Figure 1 b).^[12]^ To prove the feasibility of this approach we measured the ^13^C12‐^13^C15 distance in free retinal using SR26 build‐up experiments. We determined the distance from fitting the experimental data to SR26 build‐up curves generated using the Simpson software (Figure 3 a).^[17]^ The curves differ significantly from each other and the fitted distances correspond well to the expected distances in both molecules. We repeated the experiment on ChR2^470^ (Figure S3). A sufficient signal‐to‐noise ratio was difficult to obtain in this experiment and is an even greater challenge in the experiment with the illuminated samples as the signal intensity is split between different states. Therefore, based on the free retinal build‐up curves we picked only four different build‐up time points (3, 5, 7 and 9 ms) by which the all‐*trans* and 13‐*cis* curves can be clearly distinguished. In this way we could record the SR26 spectra with a sufficient signal‐to‐noise ratio to deconvolute the spectra and obtain the signal intensities (for the deconvolution see Figure S4 and supplementary section 1 for the details of the data analysis).

Figure 3 b shows the SR26 build‐up curves of the ^13^C12 signal. The ChR2^470^ signal in the dark sample as well as the ChR2^470^ signal that remains in the 470 nm illumination at 170 K sample showed similar build‐up curves which were fitted to similar distances corresponding to the all‐*trans* configuration as expected. In contrast the curve of ^13^C12‐P_1_^500^ in the 170 K illuminated sample looked very different and fitted to 3.1 Å which is in good agreement with the expected 13‐*cis* configuration. The ^13^C12‐P_4_^480^ curve in the sample illuminated at 245 K looks similar to the ^13^C12‐P_1_^500^ curve and unambiguously confirms the 13‐*cis* configuration of the chromophore in this state. The P_x_^13^C12 signal overlaps with the natural abundance lipid signal and the build‐up curve of this signal cannot be easily interpreted. For analysis of the P_x_ state, we therefore resorted to analysis of the ^13^C15 signal. Figure 3 c shows the results for ^13^C15‐ChR2^470^ in the dark sample and ^13^C15‐P_4_^480^ and ^13^C15‐P_x_ in the sample illuminated at 245 K. In agreement with the results on ^13^C12, ChR2^470^ and P_4_^480^ curves are fitted to 3.7 Å and 3.0 Å confirming the all‐*trans* and 13‐*cis* configuration, respectively. Interestingly, the ^13^C15‐P_x_ build‐up curve fits to a distance of 3.4 Å which is neither in agreement with a planer all‐*trans* nor a planar 13‐*cis* configuration. Thus, the retinal is in a twisted configuration which also explains the intermediate position of the chemical shift of the ^13^C12 signal.

The observed 13‐*cis*,15‐*syn* configuration of P_4_^480^ shows that the desensitized state does not correspond to the all‐*trans*,15‐*anti* O‐state in the BR photocycle. Thus, to revert to the dark state, the chromophore configuration has to change which could explain the long lifetime of the desensitized state. Interestingly, the observed configuration is the same as the population that arises during thermal equilibration of Bacteriorhodopsin in the dark.^[13c]^ The twisted chromophore structure of the P_x_ state has not been observed so far in any microbial rhodopsin. We speculate that the photoreaction of P_4_^480^ leads to a C13 *cis*‐*trans* isomerization similar to the initial photoreaction. This would result in a 13‐*anti*,15‐*syn* configuration in P_x_. The shape of the chromophore thus resembles somewhat the K‐like P_1_^500^ state. The energy stored in the P_1_^500^ state is transferred to the protein resulting in Schiff base deprotonation. For P_x_ no such reaction chain follows and the steric hindrances caused by this configuration can only be released by distorting the chromophore. Further experimental evidence is needed to support this idea.

**Figure 4:**
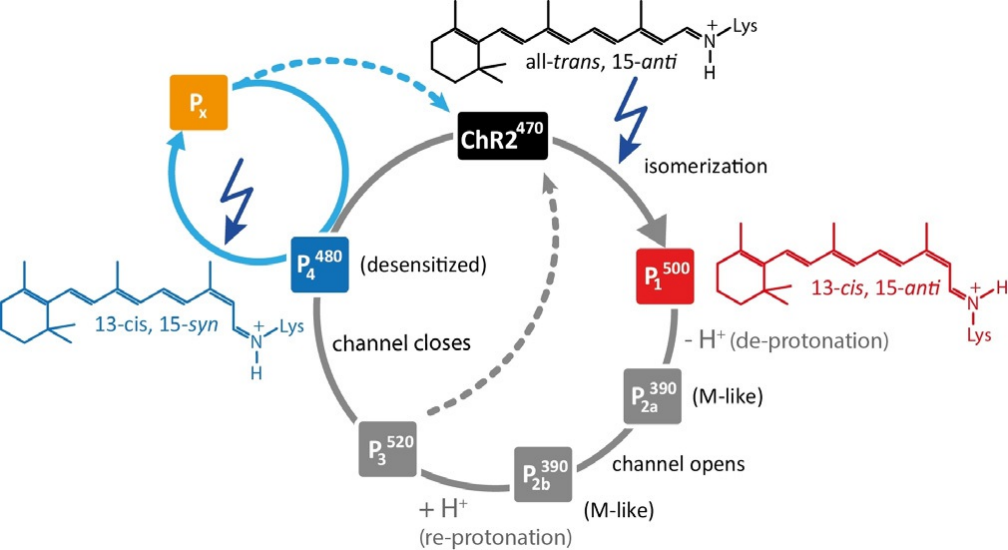
Revised photocycle of ChR2. ChR2^470^ has an all‐*trans*,15‐*anti* chromophore and illumination results in the 13‐*cis*,15‐*anti* P_1_^500^ state. The deprotonated P_2a_^390^, P_2b_^390^ and open channel P_3_^520^ states follow but no trapping protocol exists for these states. A 13‐*cis*,15‐*syn* chromophore is observed in the desensitized P_4_^480^ state which is populated after channel closure. P_4_^480^ itself is photoactive and we term its photo product P_x_. P_x_ contains a twisted chromophore structure.

In addition to time resolved experiments, cryo‐trapping photointermediates using different illumination and temperature schemes can provide insights into the sequence of photointermediates in a photo cycle. We therefore compared our observations with the different models postulated (Figure S1). We observe that the only photo product obtained below 200 K is P_1_^500^. As the signal‐to‐noise ratio of the spectra in Figure 2 b–d) is limited due to the long distance of the atoms used for double quantum filtering, we replotted the double quantum filtered spectra on [14,15‐^13^C_2_]‐retinal‐ChR2 from our previous work to unambiguously show that the P_4_^480^ photointermediate is not present in samples illuminated below 200 K (Figure S6).^[5]^ This is in contrast to the idea that P_4_^480^ is also a direct product of the photo reaction. To generate P_4_^480^ additional thermal energy is needed and we could trap the state when illuminating at 245 K. We have shown that P_4_^480^ can also be reached by thermal relaxation of P_1_^500^ (without further illumination). This again is in contrast to the proposal that P_4_^480^ is generated directly from the dark state. Our observations agree with a photocycle model where P_4_^480^ is generated at a later state in the photocycle, for example, during the open state decay as suggested before.^[7]^ We did not discus these observation in detail in our previous work,^[5]^ as the results were in agreement with the photocycle models at that time and a model with the early P_4_^480^ state had not been postulated. To reproduce and confirm our results we performed a thermal relaxation experiment on the [12,15‐^13^C_2_]‐retinal‐ChR2 sample. The proteoliposomes were illuminated at 170 K to form the P_1_^500^ state and subsequently the sample temperature was increased to 245 K in the dark (Figure 1 d, Figure 2 e). The ^13^C12‐P_1_^500^ signal completely disappeared and instead the ^13^C12‐P_4_^480^ signal at 124.5 ppm is seen confirming that P_4_^480^ follows P_1_^500^ in the photocycle. Figure 4 shows a photocycle model that agrees with our experimental data. The ChR^470^ population in the spectrum from the thermal relaxation experiment (Figure 2 e) is increased compared to the P_1_^500^ cryo‐trapping condition (470 nm @ 170 K, Figure 2 c), showing that the open state decays not exclusively towards P_4_^480^ but also a direct shortcut to ChR2^470^ may exist (dashed grey arrow in Figure 4). It can also be expected that P_x_ can undergo a thermal conversion to the dark state (dashed blue arrow in Figure 4).

## Conclusion

In summary, for the first time, double quantum filtering of atoms at distances up to 3.7 Å has been shown in a reconstituted membrane protein. Based on this method, we have resolved the controversy of the chromophore configuration of the desensitized state of ChR2 (P_4_^480^) using solid‐state NMR spectroscopy as a readout which circumvents the assignment problems which pose a challenge to the interpretation of vibrational spectroscopy data. The P_4_^480^ chromophore is in the 13‐*cis*,15‐*syn* configuration which fits well to its long lifetime. In addition, we could show that P_4_^480^ is not directly formed after light excitation but occurs at a later state in the photocycle, probably during channel closure. P_4_^480^ itself is photoactive and we could trap and analyze its photoproduct P_x_ suggesting that it has a non‐planar chromophore structure. Here, we have shown that DNP‐enhanced solid‐state NMR spectroscopy in combination with cryo‐trapping of photointermediates reveals details of the chromophore configuration in ChR2, which are difficult to obtain by other methods. In addition, we could show that reaction pathways can be deduced from the thermal relaxation pathways of cryo‐trapped samples. Therefore, we would like to advocate the use of solid‐state NMR spectroscopy in studying photoactive proteins.

## Conflict of interest

The authors declare no conflict of interest.

## Supporting information

As a service to our authors and readers, this journal provides supporting information supplied by the authors. Such materials are peer reviewed and may be re‐organized for online delivery, but are not copy‐edited or typeset. Technical support issues arising from supporting information (other than missing files) should be addressed to the authors.

Supplementary
