# Supplementary material for: The Desensitized Channelrhodopsin‐2 Photointermediate Contains 13 ‐cis, 15 ‐syn Retinal Schiff Base
Source: Angew Chem Int Ed Engl. 2021 Jun 17;60(30):16442–7. doi: 10.1002/anie.202015797 (PMC8362212; doi:10.1002/anie.202015797)
Supplement: Supplementary file 1 — Supplementary [file ANIE-60-16442-s001.pdf]

Angewandte Chemie
Eine Zeitschrift der Gesellschaft Deutscher Chemiker

Supporting Information

The Desensitized Channelrhodopsin-2 Photointermediate Contains 13-*cis*,15-*syn* Retinal Schiff Base

Johanna Becker-Baldus, Alexander Leeder, Lynda J. Brown, Richard C. D. Brown, Christian Bamann, and Clemens Glaubitz**

anie_202015797_sm_miscellaneous_information.pdf

1. Experimental details

Retinal synthesis:

[12,15-$^{13}C_2$]-all-*trans*-retinal and [12,15-$^{13}C_2$]-13-*cis*-retinal were prepared as described previously.[1]

Protein expression, purification and reconstitution:

ChR2 from *Chlamydomonas reinhardtii* was prepared as described previously.[2] Briefly, ChR2 (1-315) with a C-terminal nine-His tag was expressed in *Pichia pastoris* in the absence of retinal. The crude membranes were incubated with 5 µM [12,15-$^{13}C_2$]-all-*trans*-retinal for 2 h on ice. The protein was solubilized with 1% [w/v] β-decyl-maltoside (DM) and purified with a Ni-NTA Sepharose column. ChR2 was then reconstituted into POPC:POPG:cholesterol [8:1:1, w/w/w] proteoliposomes resulting in a lipid to protein ratio of 3:1 [w/w].

NMR sample preparation:

a) Retinal samples

1 mg of [12,15-$^{13}C_2$]-all-*trans*-retinal and [12,15-$^{13}C_2$]-13-*cis*-retinal, respectively, were dissolved in 1,1,2,2-tetrachlorethan containing 10 mM btbK as polarizing agent. The samples were packed in 3.2 mm ZrO_2 rotors.

b) Channelrhodopsin sample

The [12,15-$^{13}C_2$]-all-*trans*-retinal-ChR2 proteoliposomes were pelleted by ultracentrifugation for an hour. The sample contained around 1 mg of protein. The resulting pellet was then incubated over night with DNP buffer (20 mM AMUPOL, 30% [v/v] d_8-glycerol, 60% [v/v] D_2O and 10% H_2O [v/v]).[3] The pellet was then packed into a 3.2 mm sapphire NMR rotor and briefly spun at room temperature with a few kHz to evenly distribute the sample on the outer surface of the transparent rotor to enable rotation at cryogenic temperatures and to facilitated light penetration to the sample.

NMR experiments:

All NMR experiments were performed using a Bruker Avance II 400 MHz NMR spectrometer equipped with a 3.2 mm Cryo-MAS probe with a waveguide for transmission of microwaves. The probe was modified to enable illumination of the spinning rotor.[2a] A 263 GHz Bruker Gyrotron was used as microwave source and the microwave power at the probe was around 10 W. Enhancements from comparing experiments with and without microwave were around 16 for the retinal/tetrachloethan samples and 50 for the proteoliposome sample, respectively.

For illumination a 470 nm high-power LED from Mightex was connected to the internal light guide of the 3.2 mm Cryo-MAS probe. The nominal output power of the LED was 3.3 W but losses occur due to coupling from the LED to an external lightguide and then to an internal light guide. In addition, the spinning rotor will also scatter some light. It is therefore difficult to estimate how much light entered the sample. Full ground state depletion can be achieved at illumination at 245 K (Figure 2d) showing that sufficient light is applied for compete photoconversion. P_1^{500} was trapped by illumination at 170 K for around 20 min. A P_4^{480} and a P_x mixture was generated by illumination at 245 K for around 20 min. P_4^{480} could also be obtained by increasing the temperature of the sample after illumination at 170 K to 245 K in the dark. After each illumination scheme, the light was turned off and the sample temperature was reduced to 105 K for recording of the DNP enhanced NMR spectra.

8 kHz magic angle sample spinning was used for all experiments and microwave irradiation was on during all measurements except for the reference spectra recorded to determine the DNP-

enhancement. ^{13}C CP experiments were performed with a ramp on the proton channel and 100 kHz Spinal64 decoupling. Double quantum filtering was done using SR26 recoupling with 52 kHz on the ^{13}C channel while ^{1}H decoupling was set to around 105 kHz.[4] Referencing was done indirectly to DSS using the CH_2 signal of d_8-glycerol at 64.8 ppm.

The cross polarization 1D spectra shown in Figure 2 were recorded using 1024 scans. The SR26 double quantum filtered spectra shown in Figure 2 were recorded with a double quantum build-up and reconversion time of 5 ms and a large number of scans: 19968 (dark), 26624 (170 K illumination), 24576 (245 K illumination) and 49152 (170 K illumination followed by a temperature increase in the dark to 245 K). The other data points for the SR26 build-up curves were recorded with the same number of scans as the 5 ms experiments. In the 13-*cis*-retinal and all-*trans*-retinal sample the double quantum efficiency was 14% and 9.5%, respectively. In the Channelrhodopsin sample only the C15 signal in the dark state has a resolved signal in the CP spectrum and enabled estimation of the double quantum efficiency, yielding around 7%. We expect the double quantum efficiencies to be slightly higher in the photointermediates with a 13-*cis* chromophore configuration due to the shorter distance. On the model system used in Kristiansen et al. the achieved double quantum efficiency was 18.8%.[4] Our slightly reduced efficiencies can be attributed reduced T_2 times due to relaxation enhancement by the DNP polarizing agent AMUPOL and maybe less efficient decoupling.

All NMR experiments were record with an acquisition time of 10 ms using 592 data points. The FIDs were zero filled to 8192 data points and multiplied with a Gaussian window function (Topspin parameters: LB = -20 Hz, GB = 0.05) prior to Fourier transformation.

<u>Simulation of build-up curves:</u>

SIMPSON was used for the simulation of the SR26 build-up curves using varying ^{13}C-^{13}C dipolar coupling constants.[5] CSA parameters were taken from Smith et al..[6] The input file with all the relevant parameters is given in section 4 of this SI.

<u>Data Analysis:</u>

Data analysis was done using OriginPro 2017. All spectra from each SR26 build-up were added together and deconvoluted using Gaussian line shapes to obtain the average peak position and the linewidth of the signals (Figure S3). Using this position and linewidth the single experiments were fitted leaving the intensity of the signal as the only variable. All error bars given in the figures correspond to a 95% confidence interval. The obtained build-up curves were then fitted to the simulated build-up curved using an additional decay function to account for the apparent T_2 relaxation during the recoupling sequence. The results depend on the choice of T_2. For the retinal in tetrachlorethan samples T_2 (6.8 ms) was obtained from fitting the cis-retinal curve and then also used for analysis of the all-trans-retinal data, as T_2 was expected to be very similar in the two samples. In a similar manner T_2 was determined for the ChR2-proteoliposom sample. A build-up curve in the dark was recorded with 10 different build-up times (3, 4, 5, 6, 7, 8, 9, 10, 12 ms) and the intensities of C12 and C15 were added to further improve the signal-to-noise ration. The dipolar interaction was then fixed to -150 Hz (3.7 Å) as expected in the ground state. Fitting yielded a T_2 time of 5.21 ms which was then used in all subsequent simulations to obtain the dipolar couplings which was then converted to internuclear distances (Figure S1). The error of the dipolar coupling of the best fitting curves was estimated to be within ± 25 Hz (Figure S2).

2. Assignment of ^{13}C12 and ^{13}C15 in the SR26 spectra shown in Figure 2b-e)

The ^{13}C15 chemical shift in the dark state has been assigned previously and is easily identified at 166.5 ppm.[2a] The only other signal remaining in the dark spectrum is therefore assigned to ^{13}C12. After illuminating the sample at 170 K with blue (470 nm) light, the intensity of the ^{13}C12-ChR2^{470} signal decreases and a new signal appears at 123.2 ppm. In our previous paper we showed that only the first photointermediate P_1^{500} is accessible at temperatures below 200 K and thus we assign the signal to ^{13}C12-P_1^{500}. ^{13}C15-ChR2^{470} and ^{13}C15-P_1^{500} are not resolved. As observed earlier and also in other retinal proteins when trapping the first intermediate some dark state population always remains as the photo reaction is reversible.[7] In contrast, illumination at 245 K results in a depopulation of the dark state (no signal at 136.8 ppm) and two new ^{13}C12-signals and two ^{13}C15-signals are observed. The more intense pair of signals is assigned to P_4^{480} and the weaker one to its photoproduct P_x based on our previous work. There we could show that a species in the sample illuminated at 245 K is the same as the one that occurs when freeze quenching the sample which enriches the long lived P_4^{480} state. This assignment is confirmed by the spectrum recorded after illumination at 170 K followed by relaxation in the dark at 245 K (Figure 2e). This spectrum also shows the ^{13}C12-P_4^{480} and ^{13}C15-P_4^{480} signals as well as ChR2^{470} signals whereas the signals of the photoproduct P_x are not observed as after the generation of P_1^{500} no further photons were applied in this experiment. The assignment is summarized in Table 1 of the main text.

3. Supplementary Figures

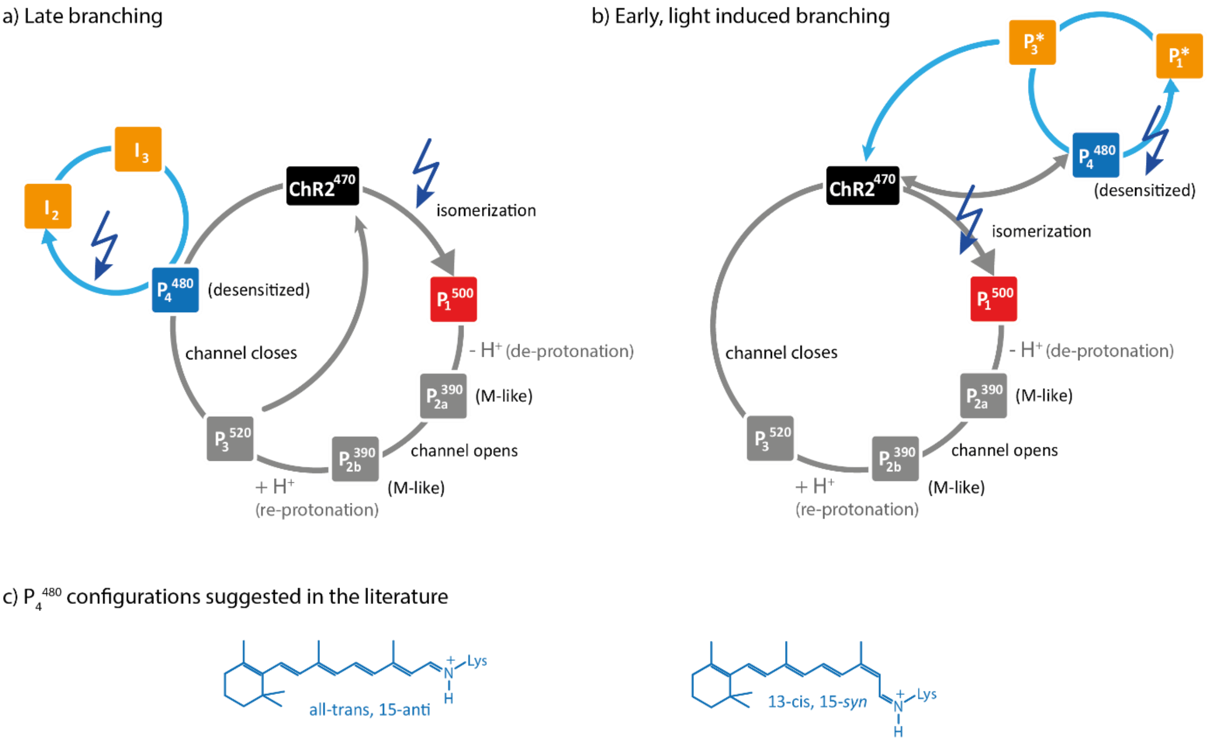

Figure S1: a) Schematic representation of the photocycle model suggested by Saita et al.[8] **b)** Schematic representation of the photocycle model suggested by Kuhne et al.[9] **c)** P_4^{480} configurations suggested in the literature.[9-10]

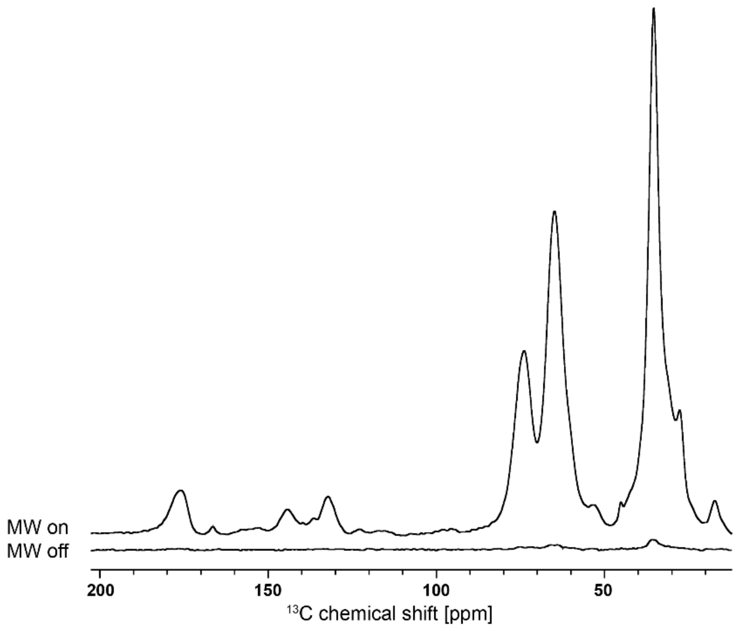

Figure S2: Spectra of [12,15-$^{13}C_2$]-all-trans-retinal-ChR2 in the dark with and without microwave irradiation recorded with 128 scans each, showing a signal enhancement around 50.

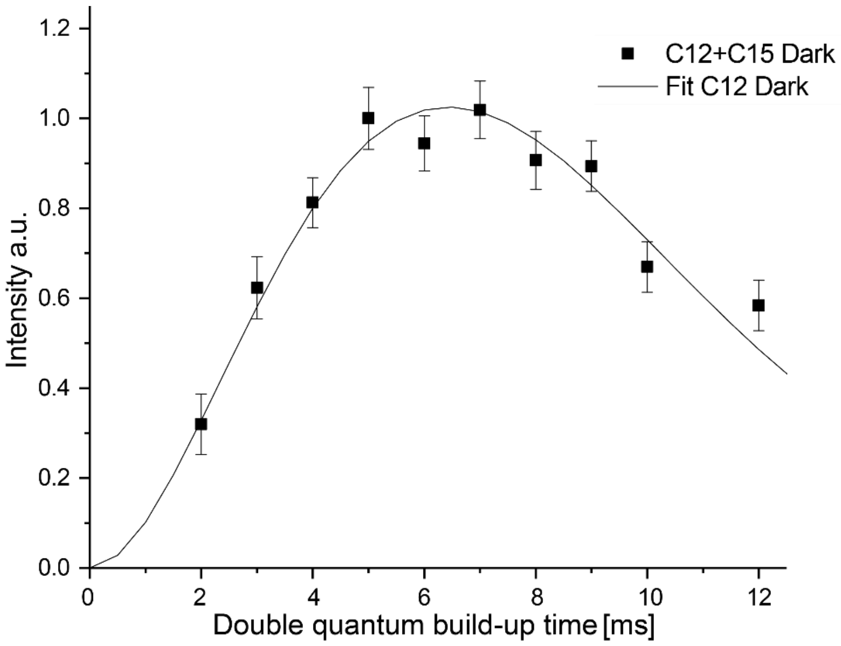

Figure S3: SR26 build-up curve of ChR2^{470}. The shown intensity corresponds to the sum of the ^{13}C12 and ^{13}C15 signals. Based on the all-*trans* conformation in the dark state, the dipolar coupling was fixed to -150 Hz which corresponds to a distance of 3.7 Å. The apparent T_2 time needed to fit this data was 5.21 ms and was used for all subsequent fits on the ChR2 sample.

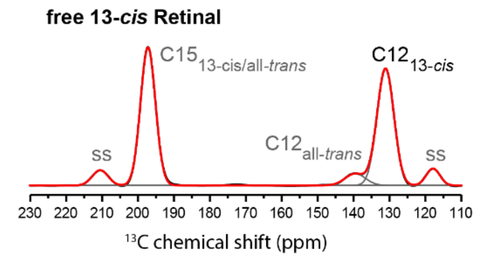

position (ppm)	line width (ppm)	assignment
210.6	5.1	sideband
197.2	4.9	C15 (13-*cis*) + C15 (all-*trans*)
139.5	6.6	C12 (all-*trans*)
**131.1**	**5.5**	**C12 (13-*cis*)**
117.9	4.4	sideband

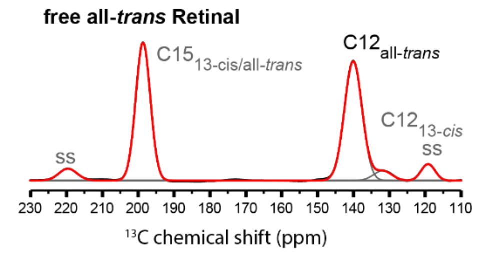

position (ppm)	line width (ppm)	assignment
219.6	6.0	sideband
198.6	4.8	C15 (13-*trans*) + C15 (all-*cis*)
**140.1**	**5.7**	**C12 (all-trans)**
131.9	5.9	C12 (13-*cis*)
119.2	4.4	sideband

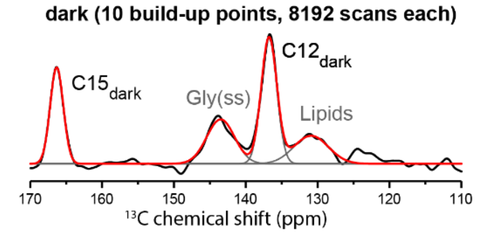

position (ppm)	line width (ppm)	assignment
**166.3**	**2.2**	**C15-ChR2^{470}**
143.5	3.87	glycerol sideband
**136.8**	**2.6**	**C12 -ChR2^{470}**
130.9	4.8	lipid background

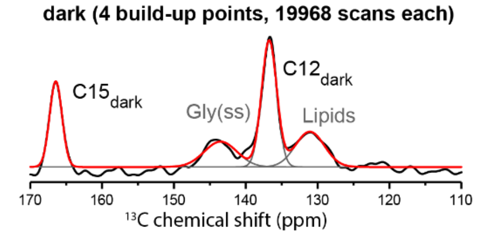

position (ppm)	line width (ppm)	assignment
**166.5**	**2.0**	**C15-ChR2^{470}**
143.5	5.4	glycerol sideband
**136.8**	**2.5**	**C12 -ChR2^{470}**
131.1	4.8	lipid background

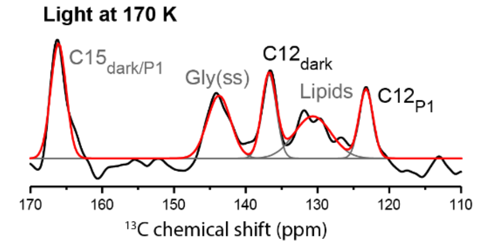

position (ppm)	line width (ppm)	assignment
166.1	2.5	C15-ChR2^{470} + C15-P_1^{500}
143.7	3.8	glycerol sideband
**136.8**	**2.4**	**C12-ChR2^{470}**
130.7	6.0	lipid background
**123.2**	**2.3**	**C12-P_1^{500}**

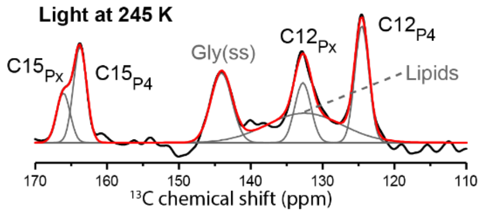

position (ppm)	line width (ppm)	assignment
**166.1**	**2.2**	**C15-Px**
**163.8**	**2.2**	**C15-P_4^{480}**
144.1	3.4	glycerol sideband
132.8	2.6	C12-Px
132.6	12.3	lipid background
**124.5**	**2.3**	**C12-P_4^{480}**

Figure S4: Peak position and line width needed for fitting the individual spectra of the SR26 build-up curves were obtained by adding all spectra of the respective SR26 build-up experiment followed by deconvolution. Deconvolution of free [12,15-$^{13}C_2$]-13-*cis*-retinal, free [12,15-$^{13}C_2$]-all-*trans*-retinal and [12,15-$^{13}C_2$]- all-*trans*-retinal-ChR2 recorded under different illumination schemes as indicated in the individual figures are shown, experimental data in black, cumulative fit in red, single signals in grey. All line shapes were assumed to be Gaussian. Chemical shifts and line width obtained from the deconvolutions are given in the respective tables. SR26 build-up curves were analyzed for the signals given in bold.

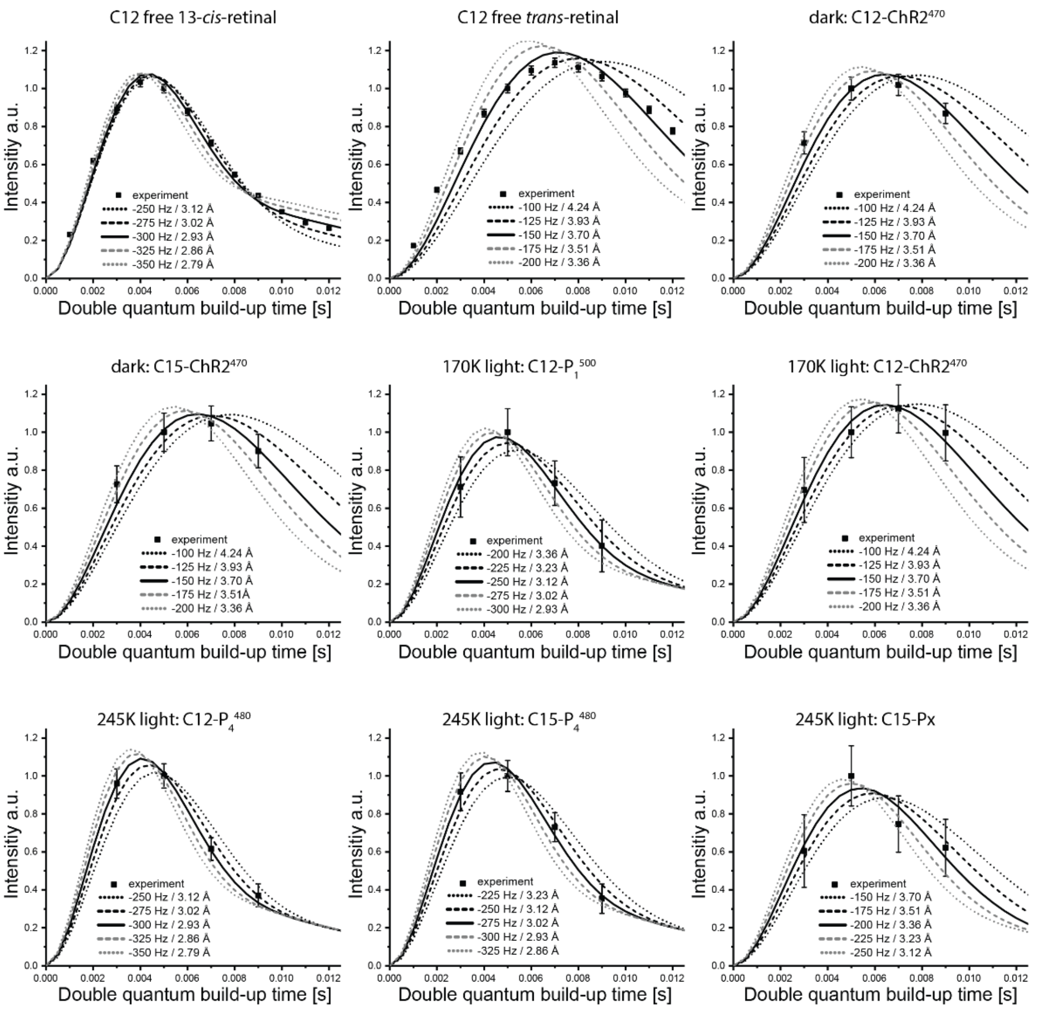

Figure S5: Experimental SR26 build-up curves with the best fit curves and curves deviating by a difference in the dipolar coupling constant of 25 Hz and 50 Hz illustrating that the error range of the dipolar coupling is ± 25 Hz.

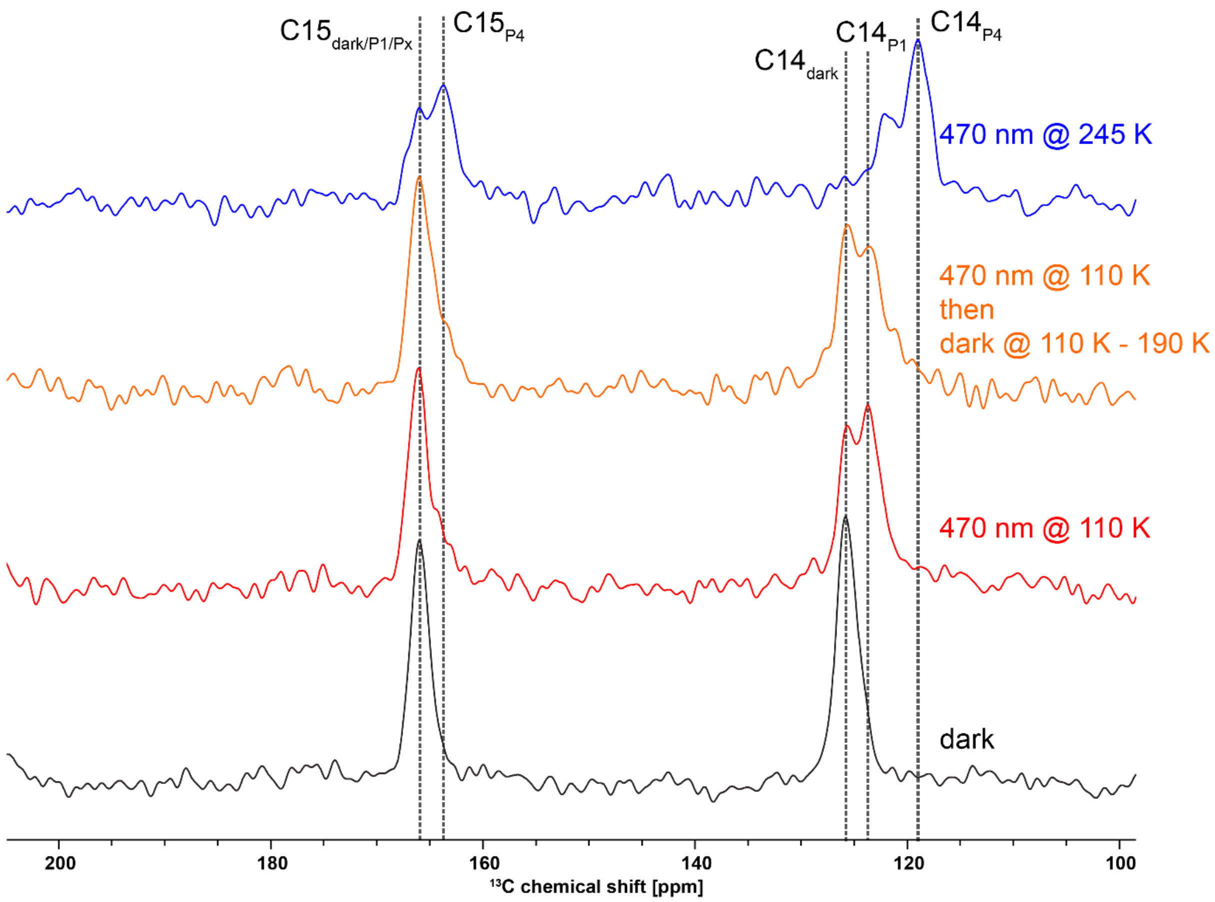

Figure S6: ^{13}C DQF spectra of [14,15-$^{13}C_2$]-all-*trans*-retinal-ChR2 recorded using different illumination schemes. The spectra are replotted from our previous work on channelrhodopsin-2 and are shown here to support the interpretation of the [12,15-$^{13}C_2$]-all-*trans*-retinal-ChR2 spectra reported here.[2a] The spectra were recorded using POST-C7 double quantum filtering.[11] P_1^{500} is seen in the spectra illuminated at 100 K (red) and relaxed at 190 K (orange) but they do not show the characteristic P_4^{480} C14 signal at 119.3 ppm which is seen in the spectrum illuminated at 245 K (blue). The C15 peak is broadened in the spectra containing P_1^{500} due to the P_1^{500}-C15 signal which overlaps with the dark ChR2^{470}-C15 signal. The data show that no intensity at the P_4^{480} chemical shift of 119.3 ppm occurs upon illumination or thermal relaxation below 200 K, proving that the initial photoreaction does not create the P_4^{480} state as discussed in the main text.

4. **Simpson input script**

```
# Excitation curve for the SR26 pulse sequence

spinsys {
  channels 13C
  nuclei 13C 13C
      # Spin-1: chem. shift, anisotropy, assymmetry, alpha, beta, gamma
      # syntax: 1000=1000Hz, 10p=10ppm
      shift 1 -20 -72p 0.93 0 0 0
      # Spin-2
      shift 2  20 -95p 0.54 0 0 0
      # Coupling between spin 1 and spin 2
  dipole 1 2 -50 0 0 0
}

par {
      proton_frequency 400.2e6
  spin_rate      8000
  crystal_file    rep100
  gamma_angles    8
  np          1048
  start_operator  I1z
  detect_operator  -I2z
  verbose       1111
  sw          6.5*spin_rate/26
}

proc pulseq {} {
  global par

  maxdt 3.0

  set rf [expr 6.5*$par(spin_rate)]
  set t90 [expr 0.25e6/$rf]
      set phaselist1 [list 76.15 76.15 76.15 76.15 76.15 76.15 76.15 76.15 76.15 76.15 76.15
      76.15 76.15 283.85 283.85 283.85 283.85 283.85 283.85 283.85 283.85 283.85 283.85 283.85
      283.85 283.85 103.85 103.85 103.85 103.85 103.85 103.85 103.85 103.85 103.85 103.85 103.85
      103.85 103.85 256.15 256.15 256.15 256.15 256.15 256.15 256.15 256.15 256.15 256.15 256.15
      256.15 256.15]
      set phaselist2 [list 256.15 256.15 256.15 256.15 256.15 256.15 256.15 256.15 256.15 256.15
      256.15 256.15 256.15 103.85 103.85 103.85 103.85 103.85 103.85 103.85 103.85 103.85 103.85
      103.85 103.85 103.85 283.85 283.85 283.85 283.85 283.85 283.85 283.85 283.85 283.85 283.85
      283.85 283.85 283.85 76.15 76.15 76.15 76.15 76.15 76.15 76.15 76.15 76.15 76.15 76.15
      76.15 76.15]
      set phaselist3 [list 283.85 283.85 283.85 283.85 283.85 283.85 283.85 283.85 283.85 283.85
      283.85 283.85 283.85 76.15 76.15 76.15 76.15 76.15 76.15 76.15 76.15 76.15 76.15 76.15
      76.15 76.15 256.15 256.15 256.15 256.15 256.15 256.15 256.15 256.15 256.15 256.15 256.15
      256.15 256.15 103.85 103.85 103.85 103.85 103.85 103.85 103.85 103.85 103.85 103.85 103.85
      103.85 103.85]
      set phaselist4 [list 103.85 103.85 103.85 103.85 103.85 103.85 103.85 103.85 103.85 103.85
      103.85 103.85 103.85 256.15 256.15 256.15 256.15 256.15 256.15 256.15 256.15 256.15 256.15
      256.15 256.15 256.15 76.15 76.15 76.15 76.15 76.15 76.15 76.15 76.15 76.15 76.15 76.15
      76.15 76.15 283.85 283.85 283.85 283.85 283.85 283.85 283.85 283.85 283.85 283.85 283.85
      283.85 283.85]


  for {set i 1} {$i <= 52} {incr i} {
    reset [expr ($i-1) * 8 * $t90 ]
    pulse $t90 $rf [lindex $phaselist1 [expr ($i-1)]]
    pulse [expr 3.0*$t90] $rf [lindex $phaselist2 [expr ($i-1)]]
          pulse $t90 $rf [lindex $phaselist3 [expr ($i-1)]]
    pulse [expr 3.0*$t90] $rf [lindex $phaselist4 [expr ($i-1)]]
    store $i
  }

  reset
  acq
  for {set i 1} {$i < $par(np)} {incr i} {
    prop [expr (($i-1) % 52) + 1]
          if {[expr ($i % 26) == 0]} {acq}
  }
}

proc main {} {
 global par
 set f [fsimpson]
 fsave $f $par(name).fid
 funload $f

}
```

References

[1] A. J. Leeder, L. J. Brown, J. Becker-Baldus, M. Mehler, C. Glaubitz, R. C. D. Brown, *Journal of labelled compounds & radiopharmaceuticals* **2018**, *61*, 922-933.

[2] a) J. Becker-Baldus, C. Bamann, K. Saxena, H. Gustmann, L. J. Brown, R. C. Brown, C. Reiter, E. Bamberg, J. Wachtveitl, H. Schwalbe, C. Glaubitz, *Proceedings of the National Academy of Sciences of the United States of America* **2015**, *112*, 9896-9901; b) C. Bamann, R. Gueta, S. Kleinlogel, G. Nagel, E. Bamberg, *Biochemistry* **2010**, *49*, 267-278.

[3] J. Becker-Baldus, C. Glaubitz, *eMagRes* **2018**, 79-92.

[4] P. E. Kristiansen, M. Carravetta, W. C. Lai, M. H. Levitt, *Chem. Phys. Lett.* **2004**, *390*, 1-7.

[5] M. Bak, J. T. Rasmussen, N. C. Nielsen, *Journal of Magnetic Resonance* **2000**, *147*, 296-330.

[6] S. O. Smith, H. J. M. De Groot, R. Gebhard, J. M. L. Courtin, J. Lugtenburg, J. Herzfeld, R. G. Griffin, *Biochemistry* **1989**, *28*, 8897-8904.

[7] a) V. S. Bajaj, M. L. Mak-Jurkauskas, M. Belenky, J. Herzfeld, R. G. Griffin, *Proceedings of the National Academy of Sciences* **2009**, *106*, 9244-9249; b) M. Mehler, C. E. Eckert, A. J. Leeder, J. Kaur, T. Fischer, N. Kubatova, L. J. Brown, R. C. D. Brown, J. Becker-Baldus, J. Wachtveitl, C. Glaubitz, *J Am Chem Soc* **2017**, *139*, 16143-16153.

[8] M. Saita, F. Pranga-Sellnau, T. Resler, R. Schlesinger, J. Heberle, V. A. Lorenz-Fonfria, *J Am Chem Soc* **2018**, *140*, 9899-9903.

[9] J. Kuhne, J. Vierock, S. A. Tennigkeit, M. A. Dreier, J. Wietek, D. Petersen, K. Gavriljuk, S. F. El-Mashtoly, P. Hegemann, K. Gerwert, *Proceedings of the National Academy of Sciences of the United States of America* **2019**, *116*, 9380-9389.

[10] V. A. Lorenz-Fonfria, B. J. Schultz, T. Resler, R. Schlesinger, C. Bamann, E. Bamberg, J. Heberle, *J Am Chem Soc* **2015**, *137*, 1850-1861.

[11] M. Hohwy, H. J. Jakobsen, M. Edén, M. H. Levitt, N. C. Nielsen, *The Journal of Chemical Physics* **1998**, *108*.
